# Antenatal care utilization increase the odds of women knowledge on neonatal danger sign: a community-based study, eastern Ethiopia

**DOI:** 10.1186/s13104-018-3957-6

**Published:** 2018-11-29

**Authors:** Tesfaye Assebe Yadeta

**Affiliations:** School of Nursing and Midwifery, College of Health and Medical Sciences, Haramaya University, P.O.B. 235, Harar, Ethiopia

**Keywords:** Neonatal mortality, Haramaya, District, Danger signs, East, Ethiopia

## Abstract

**Objective:**

This study, aimed to determine women knowledge on key neonatal dander sign and associated factors among women recently gave birth in eastern Ethiopia.

**Results:**

Of the 757 women interviewed, fever was reported as a neonatal danger sign by 255 (33.7%) followed by poor sucking (24.8%), difficulty breathing (23.5%), convulsion (16.0%), lethargy (12.9%), a very small baby (11.8%) and hypothermia (2.9%). Overall 9.38% listed four or more danger signs spontaneously. Attending at least one antenatal care visit [AOR = 2.83; 95% CI (1.62, 4.93)], and giving birth at health facilities [AOR = 3.31; 95% CI (1.67, 6.53)] were significantly associated with knowledge of neonatal danger signs.

## Introduction

Danger signs are signs that can be easily identified by non-clinical personnel including the mother. The four neonatal key danger sign are convulsions/spasms/rigidity, difficult/fast breathing, very small baby, and lethargy/unconsciousness. The danger signs were selected as key because they are common, easy to recognize, and associated with a potentially severe problem [1]. Women knowledge of neonatal key danger sign is crucial to influence their decisions to seek immediate health care for their sick neonate [2, 3].

Women knowledge of neonatal danger signs was vary from country to country and across region. In sub Saharan Africa the level of women knowledge on neonate danger signs were low [4–7]. Factors associated with knowledge of women on neonate danger sign include women educational level, antenatal care visit, health facilities delivery, post natal care utilization, accompaniment by spouse [5–8].

In Ethiopia, neonatal mortality is still high at 25 deaths per 1000 live births. Maternal and neonatal health care seeking are among the lowest in the world, 32% of women attend 4 or more antenatal visits, 16% of women give birth by skilled birth attendant, 17% receive post-natal visits [9, 10]. Until the 40th day of life, neonates are considered to be strangers to the community (not human). Their deaths are not talked [11].

More than 80% of all neonatal deaths are caused by preventable and treatable, if neonates seek health care [12]. Women knowledge of neonatal key danger sign is crucial to influence their decisions to seek immediate health care for their sick neonate. Failure to seek professional help may be related to lack of knowledge of neonatal danger signs [13]. Little is known about knowledge of danger sign and associated factors in the study area. Therefore, this study aims to assessing the knowledge and associated factors women on neonate danger signs.

## Main text

### Study design, period, and setting

A community based cross sectional study was conducted to describe the current knowledge of women in June 2012. The study was conducted in East Hararge Zone, Oromia Region, eastern Ethiopia 507 km East of Addis Ababa. The district has 34 rural kebeles (neighbourhoods) and 2 urban kebeles (the smallest administrative unit in Ethiopia). Each kebeles has two health extension workers who undergo a 1 year training program and provide primary health care including basic curative and preventative services.

### Sample size determination, sampling recruitment and sampling procedure

All mothers who gave birth in the last 2 years (May 2010–April 2012) and resided in the district were the source population. Mothers residing in randomly selected kebeles were included in the sample. The sample was calculated using a single population proportion by taking awareness on neonatal danger signs of 50%, 5% margin of error, 95% confidence level, design effect of two and 5% non-response rate. A two-stage sampling method was used to select study subjects. In the first stage, we selected four kebeles out of the thirty-four kebeles using a simple random sampling method. Then women who gave birth in the last 2 years in the selected kebeles were identified using the health extension workers records (No = 1200). We used these records as the sampling frame to select the respondents using simple random sampling from a sample size of 806. The birth records are a complete list. Each kebele has two health extension workers (HEWs) who visit each household regularly. These HEWs know the number of pregnant women (including ANC status) in their catchment population, the number of women with children under 1 and between 2 and 5 years (including their children’s immunization and nutritional status).

### Data collection procedure

Data collection was undertaken using an interviewer administered structured questionnaire that was adapted from the Safe Motherhood questionnaire developed by the Maternal and Neonatal Health Program of JPHIEGO and WHO [1, 14]. The questionnaire consisted of questions to establish women’s awareness and knowledge of neonatal danger signs including convulsions or spasms or rigidity, difficult or fast breathing, very small baby, and lethargy or unconsciousness, body temperature (hypothermia, fever), and feeding (no or poor sucking). It also contained questions to gather socio-demographic data such as age, educational status, occupation status, family size; reproductive health characteristics such as gravidity, parity and antenatal follow up and health care practice. The questionnaire was prepared in English, translated to Afan Oromo and then back translated by language expertise. The questionnaire was pretested on 42 women from another district and modified accordingly. Data was collected by nurse/midwifery who had research field work experience. We provided a 2-day training course for the data collectors and supervisors. The completeness and consistency of data daily checked at the site of data collection by supervisor.

### Measurement

A knowledge score for neonatal danger signs was computed using the neonate danger signs spontaneously listed by the mothers. These signs were selected because they strongly predict neonatal mortality [1] are considered danger signs as per the conceptual framework described above [14]. All correct responses were assigned a value of 1 while 0 was given for wrong answers. All women who spontaneously mentioned the four and above danger signs were considered knowledgeable.

### Data processing and analysis

The data were entered and cleaned using EpiData version 3.1 and analysed using STATA software version 13. First descriptive statistics were computed to describe individual variables using frequencies and percentages. Binary logistic regression was fitted to assess the association between knowledge of danger signs and individual independent variables. Variables with p-value ≤ 0.2 in the bivariable analyses were considered in the multivariable logistic regression model. Adjusted odds ratio (AOR) along with 95% confidence interval was estimated to assess the strength of the association, and a p-value ≤ 0.05 was used to declare the level of statistical significance.

### Results

#### Socio-demographic and maternal health characteristics of respondents

A total of 757 (a response rate of 94%) women were interviewed with the mean age of 26.34 ± 6.62 years. Most women were Muslim (97.5%), belonged to the Oromo ethnic group (95.6%) and married (93.3%). About three in four of the women were rural residents (74%) and illiterate (72.7%). In terms of pregnancy, 29.7% of the women reported having been pregnant five times or more and 14% of the women reported experiencing abortion. A total of 323 (42.67%) women stated that they had attended at least once antenatal care (ANC) visits during their last pregnancy, of which 77 (23.83%) reported attending all the recommended four visits. Most women (86.8%) stated that they had given birth to their last baby at home and majority of the deliveries (95.8%) were normal vaginal births without complications (Table 1).

**Table 1:** Socio-demographic characteristics of women by their knowledge in Haramaya district, eastern Ethiopia, 2012 (n = 757)

Character	Total	Neonatal knowledge	
		Knowledgeable	Non-knowledgeable
Age of women in year			
≤ 25	534 (70.54%)	53 (9.93)	481 (90.07)
> 25	223 (29.46%)	18 (8.07)	205 (91.93)
Residence			
Urban	197 (26.02%)	17 (8.63)	180 (91.37)
Rural	560 (73.98%)	54 (9.64)	506 (90.36)
Ethnic group			
Oromo	724 (95.64%)	69 (9.53)	655 (90.47)
Others	33 (4.36%)	2 (6.06)	31 (93.94)
Educational status			
Literate	207 (27.34%)	26 (12.56)	181 (87.44)
Illiterate	550 (72.66%)	45 (8.18)	505 (91.82)
Occupation			
House wife	453 (59.84%)	47 (10.38)	406 (89.62)
Others	304 (40.16%)	24 (7.89)	280 (92.11)
Marital status			
Married	706 (93.26%)	64 (9.07)	642 (90.93)
Others	51 (6.74%)	7 (13.73)	44 (86.27)
Number of delivery			
1–2	291 (38.44%)	18 (6.19)	273 (93.81)
3–4	307 (40.55%)	32 (10.42)	275 (89.58)
≥ 5	159 (21.01%)	21 (13.21)	138 (86.79)
History of abortion			
Yes	106 (14.00%)	10 (9.43)	96 (90.57)
No	651 (86.00%)	61 (9.37)	590 (90.63)
Pace of delivery			
Home	657 (86.79%)	48 (7.31)	609 (92.69)
Institution	100 (13.21%)	23 (23.00)	77 (77.00)
ANC follow up			
Yes	323 (42.67%)	49 (15.17)	274 (84.83)
No	434 (57.33%)	22 (5.07)	412 (94.93)

#### Knowledge of neonatal danger signs

**Fig. 1:**
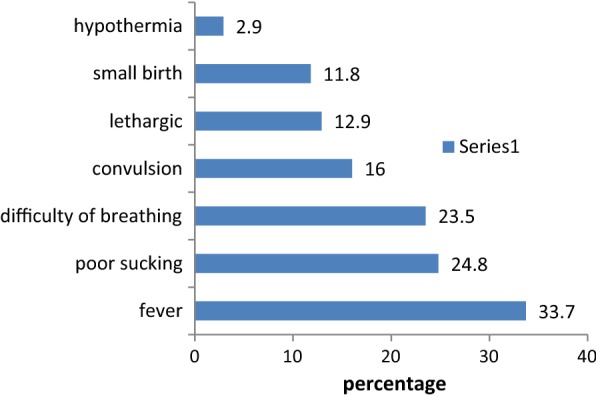
Knowledge of neonatal danger signs among women who recently gave birth in Haramaya District, eastern Ethiopia, 2012

In terms of the respondent’s knowledge the women interviewed noted awareness of seven neonatal danger signs. The most listed danger sign was fever (33.7%), followed by poor sucking of the neonate (24.8%). Overall 9.38% listed four or more danger signs spontaneously (Fig. 1).

#### Factors associated with knowledge of danger signs

The multivariable logistic regression analysis revealed antenatal follow up [AOR = 2.83; 95% CI (1.62, 4.93)] and had no given birth in a health facility [AOR = 3.31; 95% CI (1.67, 6.53)] were significantly associated with maternal knowledge of neonate danger signs (Table 2).

**Table 2:** Predictors of good knowledge about neonatal danger signs among women in Haramaya district, eastern Ethiopia, 2012

Character	Crudes OR		Adjusted OR	
	OR	95% CI	OR	95% CI
Age of women in year				
≤ 25	1.25	0.71, 2.19	1.75	0.92, 3.32
> 25	1		1	
Residence				
Urban	1.12	0.63, 2.00	2.36	0.51, 10.79
Rural	1		1	
Educational status				
Literate	1		1	
Illiterate	0.62	0.37, 1.03	0.99	0.50, 1.97
Occupation				
House wife	1		1	
Others	1.35	0.80, 2.25	1.56	0.86, 2.81
Marital status				
Married	1		1	
Others	0.62	0.27, 1.44	0.59	0.20, 1.74
History of abortion				
Yes	1		1	
No	0.99	0.49, 2.00	1.30	0.52, 3.20
ANC follow up				
No	1		1	
Yes	3.34	1.97, 5.66***	2.83	1.62, 4.93***
Pace of delivery				
Home	1		1	
Institution	3.78	2.18, 6.57***	3.31	1.67, 6.53**

* ANC* antenatal care;* OR* odds ratio

***significant at p < 0.001; **significant at p < 0.05

### Discussion

In this study, women knowledge of neonatal danger signs was 9.38%. Factors that significantly associated to women knowledge of neonatal danger signs were Women with at least one ANC follow up and giving birth at health facilities.

This study found that, only 9.38% of the women interviewed knew four or more neonatal danger signs. Women’s knowledge of neonatal danger signs varies in sub Saharan Africa. In Uganda only 14.8% could name at least two danger signs [5]. In Nigeria, the percentage of mothers who were able to recognize four or more neonatal danger signs was 30.3% [15]. In Kenya 15.5% of mothers identified four or more neonatal danger signs [16]. Women knowledge of neonatal danger sign was variable, the reason may be due to difference in socio-demographic, economic, health care utilization and women educational status [17].

Women knowledge of neonatal key danger sign is crucial to influence their decisions to seek immediate health care for their sick neonate. Failure to seek professional help may be related to lack of knowledge of neonatal danger signs [2]. To achieve sustainable development goal of their neonatal mortality reduction program planner and health facilities found in the country need to include information dissemination of neonatal danger into their maternal and neonatal health care strategies.

In this study, we found that mothers who visited of at least one ANC follow up and give birth at health facility were significantly associated with, knowledge of neonatal danger sign which is consistent with the findings of studies conducted elsewhere [5]. Counselling of mother on neonatal danger sign during ANC, delivery and PNC is one component of health care. However, ANC follow up and health facilities delivery was very low. In this study only 19.0% pregnant women for 4 visits while the proportion of women giving birth at health facilities were 10%. Urgent effective intervention is needed to improve the coverage of ANC follow up and health facilities delivery in their maternal and neonatal reduction strategy [18].

The low utilization of health services together with low awareness of neonatal danger signs is problematic and highlights the need for major action to generate demand for health services and improve community knowledge to reduce neonatal death. The results of this study can inform current initiatives that are focused on improving maternal and newborn health care delivery in rural Ethiopia. The findings of our study serve to further emphasise the important role that HEWs must continue to play in relaying information about neonatal danger signs, as well as working to actively engage community members to encourage women and their families to seek help [19].

Community involvement in health care has been found to be a cost-effective strategy to improve maternal and neonatal survival in low-resource settings [20]. However, empowering communities to participate in health care is dependent on the capacity of that community to make decisions and the socio-cultural and health system context.

Several health promotion strategies could be used to strength efforts at community level to improve community recognition of neonatal danger signs and help-seeking behaviours. Innovative approaches noted in the literature include a mobile video show designed to improve community knowledge, attitudes, and beliefs regarding maternal and newborn health, especially care-seeking behaviour and the use of a skilled birth attendant and postnatal care [2]. Mobile health (m Health) applications such as the use of mobile phones and and tablet computers also show promise [21].

Despite the possibilities shown by such strategies, alongside improved service delivery and health worker training, further efforts will be required. Community capacity building and socio-cultural change is also needed to transform Ethiopian community attitudes and behaviours, as well as a national vital registration system to provide intelligence for health service planning. Scaling up evidence-based and novel approaches and co-ordinating these is essential to accelerate an increase in women’s awareness of the signs and symptoms of newborn illness and mobilise communities to seek medical assistance to reduce preventable neonatal death.

### Conclusions

Mother’s knowledge of neonatal danger signs appears to be low. These findings reveal an urgent need to increase women awareness of neonatal danger signs. Effort is needed to access pregnant women to antenatal care and health facilities delivery services help the women to improve their awareness.

## Limitations of the study

The use of a cross sectional design in this study did not allow for causal relationships to be established, thus the reasons why women reported certain danger signs more so than others is not known. In addition, the study assessed the knowledge of woman who gave birth in the last 2 years. Findings may therefore be affected by recall bias and the failure the woman to differentiate between the neonatal and post neonatal period. Limiting interviews to mothers who had given birth within the last 28 days might have rendered more accurate data. Furthermore, the influence of women knowledge on health seeking behaviour was not assessed using theoretical model, which help health care provider for intervention. In addition, unaccounted and residual confounding could have an effect on the association.

## Abbreviations

ANC: antenatal care
HEW: health extension workers
MaNHEP: the maternal and newborn health in Ethiopia partnership
NMR: neonatal mortality rate
WHO: World Health Organization
